# Prevalence of perinatal depression among HIV-positive women: a systematic review and meta-analysis

**DOI:** 10.1186/s12888-019-2321-2

**Published:** 2019-10-30

**Authors:** Qi-Yu Zhu, De-Sheng Huang, Jian-Da Lv, Peng Guan, Xing-Hua Bai

**Affiliations:** 1 Department of Epidemiology, School of Public Health, China Medical University, Shenyang, 110122 China; 2 Department of Mathematics, School of Fundamental Sciences, China Medical University, Shenyang, China; 3 Department of Radiation Oncology, The First Affiliated Hospital of China Medical University, Shenyang, China

**Keywords:** HIV, Perinatal depression, Pregnant women, Meta-analysis

## Abstract

**Background:**

Increasing attention has been paid to differences in the prevalence of perinatal depression by HIV status, although inconsistent results have been reported. The aim of this systematic review and meta-analysis was to assess the relationship between perinatal depression and HIV infection. A comprehensive meta-analysis of comparative studies comparing the prevalence of antenatal or postnatal depression between HIV-infected women and HIV-negative controls was conducted.

**Methods:**

Studies were identified through PubMed/Medline, Scopus, Web of Science, Cochrane Library, Embase and PsycINFO, and the reading of complementary references in August 2019. Subgroup analyses were performed for anticipated explanation of heterogeneity using methodological quality and pre-defined study characteristics, including study design, geographical location and depression screening tools for depression. The overall odds ratio (OR) and mean prevalence of each group were calculated.

**Results:**

Twenty-three studies (from 21 publications), thirteen regarding antenatal depression and ten regarding postnatal depression were included, comprising 3165 subjects with HIV infection and 6518 controls. The mean prevalence of antenatal depressive symptoms in thirteen included studies was 36% (95% CI: 27, 45%) in the HIV-positive group and 26% (95% CI: 20, 32%) in the control group. The mean prevalence of postnatal depressive symptoms in ten included studies was 21% (95% CI: 14, 27%) in the HIV-positive group and 16% (95% CI: 10, 22%) in the control group. Women living with HIV have higher odds of antenatal (OR: 1.42; 95% CI: 1.12, 1.80) and postnatal depressive symptoms (OR: 1.58; 95% CI: 1.08, 2.32) compared with controls. Publication bias and moderate heterogeneity existed in the overall meta-analysis, and heterogeneity was partly explained by the subgroup analyses.

**Conclusions:**

Women with HIV infection exhibit a significantly higher OR of antenatal and postnatal depressive symptoms compared with controls. For the health of both mother and child, clinicians should be aware of the significance of depression screening before and after delivery in this particular population and take effective measures to address depression among these women.

## Background

Human immunodeficiency virus (HIV) infection has become a major contributor to the global burden of disease, with nearly 35.3 million people infected in 2012 and sub-Saharan Africa accounting for 70.8% of the infected population [1]. Fortunately, with the introduction of antiretroviral therapy (ART), the number of AIDS-related deaths has steadily declined, and the life expectancy of HIV-infected people has reliably increased. Now, in addition to finding treatments to prevent and cure HIV infection, attention has shifted toward improving HIV-infected people’s quality of life.

Psychological health is essential to quality of life. Previous studies have shown that people living with HIV are vulnerable to mental health problems, including depression, anxiety, suicidal behaviour, and substance abuse [2]. A high prevalence of depression among HIV-infected people has been reported to result in decreased life quality, low adherence to antiretroviral medications, and increased mobility [3–5]. Moreover, a cross-sectional study concluded that among people living with HIV/AIDS, women presented more severe symptoms of depression than men [6].

It has been reported that 25.9% of women who are HIV-positive intend to have children [7], and this population might face considerable psychosocial challenges because the impact of a chronic illness could be complicated by the demands of pregnancy. A systematic review that included 22 studies conducted in Africa examined the prevalence of perinatal depression in HIV-infected women and found that the weighted mean prevalence of antenatal and postnatal depression was 23.4 and 22.5%, respectively [8].

Perinatal depression, a psychiatric disorder characterized by the Diagnostic and Statistical Manual of Mental Disorders Fifth Edition (DSM-5) as a major depressive episode MDE) with peri-partum onset, i.e. symptom onset during pregnancy or in the 4 weeks following delivery, has a prevalence of 11.4 to 12.5% [9, 10]. The definition differs in research and practice, where the postpartum period has been extended to 12 months postpartum to reflect the state of the field more accurately [10]. Postnatal depression even reached a prevalence of 27.7% in one study [11]. Women with perinatal depression have increased risks of self-harm ideation, suicidal ideation, cardiovascular diseases and gestational diabetes, and depression can even induce non-adherence to ART among HIV-infected individuals [5, 12–15]. Perinatal depression usually accompanies adverse pregnancy outcomes, including premature delivery, low birth weight [16–18], and emotional, behavioural or cognitive problems in the offspring during adolescence [19]. Antenatal depression is a robust risk factor for postnatal depression, and whether the co-occurrence of this relationship with HIV infection also persists remains unknown.

Considering the adverse effects and medical demands of women with perinatal depression in the context of HIV infection, a number of studies have recently compared the prevalence of perinatal depression among HIV-infected women versus HIV-negative women and it has been found that HIV-infected women have a tendency of having perinatal depression experience [20, 21]. However, the strength of the association between HIV infection and perinatal depression remains uncertain. Antenatal and postnatal levels of depressive symptoms among HIV-infected women could afford a sound basis for the targeted treatment interventions. To the authors’ knowledge, no meta-analysis has been published on this topic. To address this gap, we conducted the present meta-analysis to compare the relative risk of antenatal and postnatal depression among HIV-positive and control subjects. Subgroup analyses of study design, study quality, depression screening tool used, and geographical location were also conducted to examine anticipated heterogeneity. The present study sought to highlight the importance of accurate depression screening and prompt intervention for perinatal HIV-positive women, thereby exerting a favourable impact on the offspring.

## Methods

### Literature search

We used the following search strategy: (antenatal OR peripartum OR perinatal OR postnatal OR postpartum) AND (depression OR mental disorder) AND HIV. A thorough literature search was conducted through PubMed/Medline, Scopus, Web of Science, Cochrane Library, Embase and PsycINFO with the language restriction of English from inception to 3 August 2019. The search results were independently screened and extracted by two investigators (QYZ and DSH), and all discrepancies were resolved by the principal investigator (PG). When data were not sufficient for meta-analysis, we tried to contact the authors through e-mail. The study was conducted in accordance with the Preferred Reporting Items for Systematic Reviews and Meta-Analyses (PRISMA) guidelines [22] (see Additional file 1).

### Inclusion and exclusion criteria

Studies were eligible for inclusion if they satisfied the following criteria: 1) both an HIV-positive group and a control group were evaluated; 2) measurement tools for depression and cut-off values were reported; 3) antenatal depression was assessed during pregnancy, and postnatal depression was assessed no more than 1 year after delivery; 4) current rates of depression were reported or could be calculated; 5) data were provided from the earliest assessment if the study was longitudinal to preclude the influence of intervention; and 6) the study subjects were not recruited specifically through the mental health system to avoid the selection bias.

### Quality assessment

The quality of each study was assessed according to a checklist previously used by Fisher et al. [23], which was initially developed by Mirza and Jenkins [24]. The checklist consists of nine items, including clear study aims, adequate sample size (or justification), representative sample (with justification), clear inclusion and exclusion criteria, valid and reliable measure of mental health, response rate reported and losses given, adequate description of data, appropriate statistical analysis and appropriate informed consent procedure; higher scores indicate higher quality. We defined scores of 0–3 as low or poor quality, 4–6 as moderate quality, and 7–9 as high or good quality. Two investigators (QYZ and DSH) independently assessed article quality, and inconsistencies were resolved by the principal investigator (PG).

### Data analysis

Antenatal and postnatal depression rates were meta-analysed using the fixed effects or random effects model, as appropriate. The Mentel-Haenszel method was adopted, and heterogeneity assessment was performed using *I*^2^ statistics, with *I*^2^ ≥ 30% considered likely to represent moderate heterogeneity; thus, a random-effects model was used when *I*^2^ ≥ 30%. The effect size was measured with odds ratios (ORs), and chi-square tests were used for the statistical significance test. It should be noted that these values measure significant differences in terms of the effect size. Depressive symptoms differences are measured by referring to scores on screening tools for antenatal and postnatal depressive symptoms. Mean prevalence was calculated with a random model based on the number of participants in each study, and 95% confidence intervals (95% CIs) were also calculated. Publication bias was assessed with visual inspection of the funnel plots. The above-mentioned statistical analyses were performed using the software Review Manager, Version 5.3 (RevMan 5.3), and Stata, Version 13.0.

### Sensitivity analysis

Considering that our meta-analysis consisted of different study types, perinatal depression was defined by various screening tools, and the studies were conducted in different geographic regions, the following subgroup analyses were also assessed the robustness of the results: methodological quality, study design, study quality, depression screening tool used, and geographical location.

## Results

### Characteristics of included studies

**Fig. 1:**
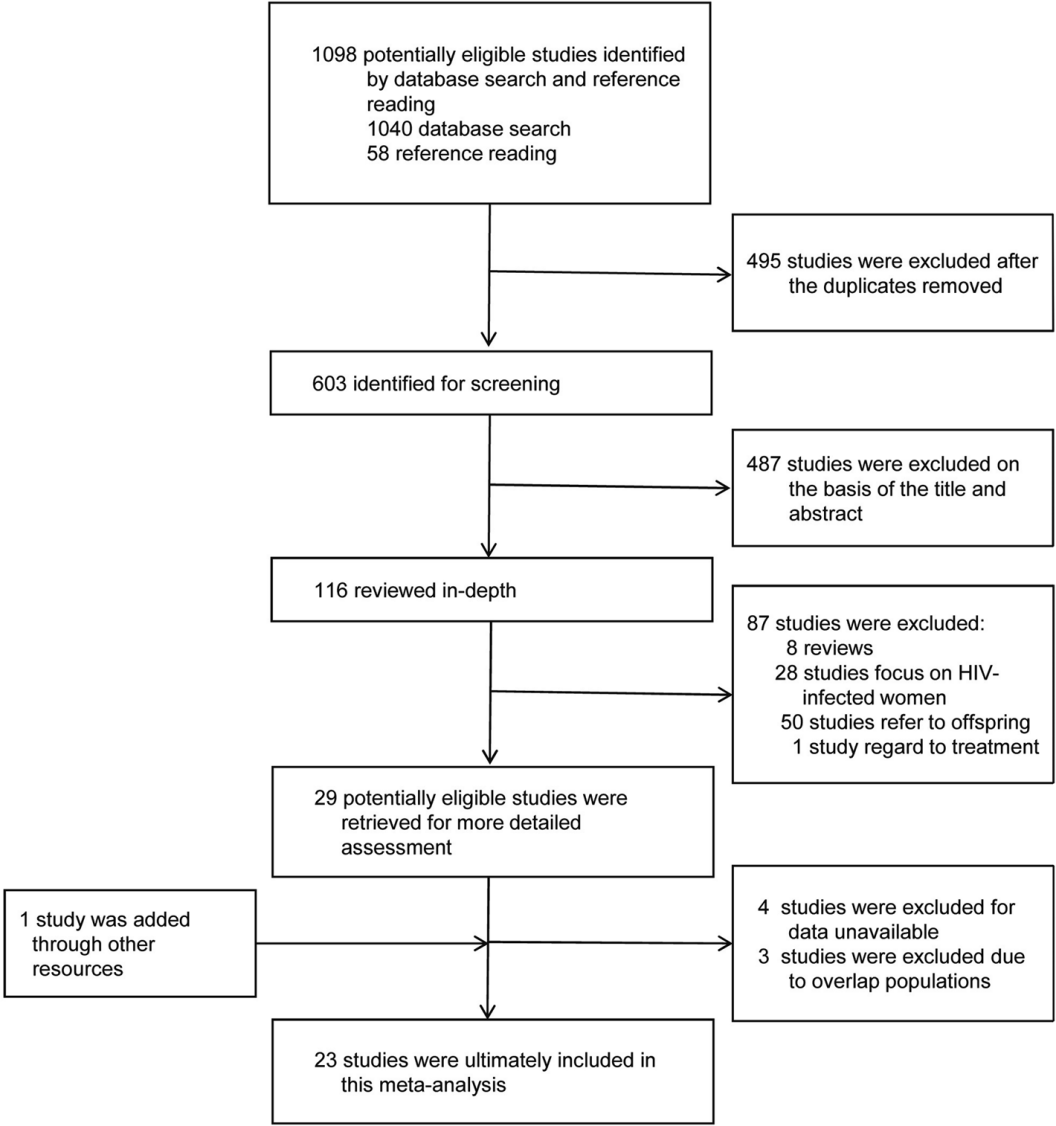
PRISMA flow diagram of study selection process of antenatal and postnatal depression in women with HIV infection compared with controls

A total of 1098 articles were found using the search strategy, and 603 publications remained after duplicates were removed. After reviewing in depth of these publications and obtaining necessary data by contacting the authors, 26 studies (24 articles) met the inclusion criteria. Due to the overlap of the study populations, only 23 studies (21 articles) were finally included in the meta-analysis [20, 21, 25–43]; thirteen studies reported antenatal depression, and ten studies reported postnatal depression (Fig. 1). One article that focused on antenatal depression defined people with unknown serostatus of HIV as controls [33] and the other studies defined HIV-negative people as controls. In total, 1520 subjects with HIV infection and 4383 controls were included in the meta-analysis of antenatal depression, and 1645 subjects living with HIV infection and 2135 controls were included in the meta-analysis of postnatal depression.

Table 1 shows the characteristics of the included studies [20, 21, 25–43]. Of the 23 studies included in our meta-analysis, eleven used the Edinburgh Postnatal Depression Scale (EPDS) for depression screening; the Center for Epidemiologic Studies Depression Scale (CES-D) was adopted by six studies; the Self-Reporting Questionnaire (SRQ) was applied in three studies; one study measured depression by the diagnostic Structured Clinical Interview for DSM-IV Axis I Disorders (SCID); one study used the Shona Symptom Questionnaire (SSQ); and the remaining study used the International Classification of Diseases 10th Revision (ICD-10) depression inventory (Major Depression Inventory). While the EPDS and SRQ are used with different cut-off values, the most frequently used cut-off for the EPDS was 13, used by nine studies, and the remaining two studies used cut-off values of 11 and 12. The analysis was first carried out without restriction of the original EPDS cut-off values, and then we also calculated the effect size when excluding the studies that adopted EPDS cut-off values below 13. For the three studies that used the SRQ, two defined 7 as the cut-off value, and the remaining one used a cut-off value of 8. Table 2 shows the methodological quality of the included studies.

**Table 1:** Characteristics of included studies of antenatal depression and postnatal depression in HIV-infected women compared with controls

Study (Author, year of publication)	Country and location	N	Type of study	Time of assessment	Instrument and cutoff value	HIV+	Controls	Number with depression in HIV+ group	Number with depression in control group
Antenatal									
Bonacquisti et al., 2014 [25]	America, Urban, 1 obstetrics/gynecology clinic	163	Case-control study	24 weeks	CES-D ≥ 16	50	113	18	41
Collin et al., 2006 [26]	Zambia, Urban, 1 antenatal clinic	181	Cross-sectional	33 weeks	SRQ-20 ≥ 7	89	92	20	20
Malqvist et al., 2016 [27]	Swaziland, Peri-urban	973	Cross-sectional	3rd trimester of pregnancy	EPDS≥13	412	561	105	117
Manikkam and Burns, 2012 [28]	South Africa, Urban, Antenatal clinic at tertiary hospital	378	Cross-sectional	28.6 weeks mean	EPDS≥13	104	201	44	66
Manongi et al., 2017 [29]	Tanzania, Semi-urban, Antenatal care clinic	1116	Cross-sectional	24 weeks	EPDS≥13	38	1078	11	117
Natamba et al., 2014 [30]	Uganda, Urban, 1 antenatal care clinic other public medical facilities	123	Case-control study	18 weeks	CES-D ≥ 16	36	87	19	25
Nydoo et al., 2017 [20]	South Africa, Urban, 2 antenatal clinics	102	Cross-sectional	1st trimester of pregnancy	EPDS≥13	40	62	2	8
Osok et al., 2018 [21]	Kenyan, Rural, Maternal child health clinic	176	Cross-sectional	Not provided	EPDS≥13	14	162	13	45
Rochat et al., 2011 [31]	South Africa, Rural, 1 primary health care clinic	109	Cross-sectional	second half of pregnancy	SCID	49	60	27	24
Rubin et al., 2011 [32]	America, Urban, 6 clinic sites	244	Longitudinal	≤10 months before delivery	CES-D ≥ 16	139	105	47	40
Stranix-Chibanda et al., 2005 [33]	Zimbabwe, Peri-urban, 3 antenatal clinics	437	Cross-sectional	3rd trimester of pregnancy	SSQ ≥ 8	62	212	12	35
Thomas et al., 2017 [34]	South Africa, Rural, 2 primary health care clinics	899	Cohort study	28–32 weeks	EPDS≥13	192	707	51	174
Tomlinson et al., 2018 [35]	South Africa, Urban, Community sample	1241	randomised controlled trial	26 weeks	EPDS>13	295	943	113	320
Postnatal									
Aaron et al., 2015 [36]	America, Urban, 1 obstetrics/gynecology clinic	162	Case-control study	6 months	CES-D > 16	49	113	15	25
Chersich et al., 2008 [37]	Kenya, Urban, a pediatric clinic in provincial hospital	500	Cross-sectional	1 year	ICD-10	54	446	2	6
Chibanda et al., 2014 [38]	Zimbabwe, Peri-urban, 2 postnatal clinics	210	Cross-sectional	6–8 weeks	EPDS≥11	31	148	14	35
Collin et al., 2006 [26]	Zambia, Urban, 1 antenatal clinic	181	Cross-sectional	7 days	SRQ-20 ≥ 7	89	92	4	6
Cyimana et al., 2010 [39]	Zambia, Urban, University teaching tertiary hospital	229	Cross-sectional	2–6 weeks	EPDS≥13	46	183	17	47
Dow et al., 2014 [40]	Malawi, Urban primary clinic and peri-urban clinic	492	Longitudinal	10–14 weeks	EPDS≥12	338	154	39	15
Mokhele et al., 2019 [41]	South Africa, N/A, Midwife Obstetric Units	1151	Cross-sectional	1 month	CES-D 10 ≥ 10	690	461	70	50
Okronipa et al., 2012 [42]	Ghana, Rural, 3 prenatal clinics	328	Cross-sectional	6 months	EPDS≥13	152	176	26	5
Rubin et al., 2011 [32]	America, Urban, 6 clinic sites	244	Longitudinal	≤12 months after delivery	CES-D ≥ 16	139	105	43	37
Stewart et al., 2008 [43]	Malawi, Rural, Child health clinic at government hospital	501	Cross-sectional	9.9 month mean	SRQ-20 ≥ 8	57	257	25	67

*Abbreviations*: *CES-D* Centre for Epidemiologic Surveys for Depression, *EPDS* Edinburgh Postnatal Depression Scale, *HIV+* HIV positive, *ICD-10* International Classification of Diseases-10 depression inventory (Major Depression Inventory), *SCID* Structured Clinical Interview for DSM-IV Axis I Disorders, *SRQ-20* Self-Reporting Questionnaire, *SSQ* Shona Symptom Questionnaire

**Table 2:** Quality evaluation of studies included in the meta-analysis

Study (first author, publication year)	Clear study aims	Adequate sample size (or justification)	Representative sample (with justification)	Clear inclusion and exclusion criteria	Measure of mental health valid and reliable	Response rate reported and losses reported	Adequate description of data	Appropriate statistical analysis	Appropriate informed consent procedure	Total score
Aaron et al. 2015 [36]	1	1	0	1	1	0	1	1	1	7
Bonacquisti et al. 2014 [25]	1	1	0	1	1	0	1	1	0	6
Chersich et al. 2008 [37]	1	1	0	1	1	1	1	1	0	7
Chibanda et al. 2014 [38]	1	0	1	1	1	0	1	1	1	7
Collin et al. 2006 [26]	1	0	0	1	1	1	1	1	1	7
Cyimana et al. 2010 [39]	1	1	1	0	1	0	1	1	1	7
Dow et al. 2014 [40]	1	0	0	0	1	1	1	1	1	6
Malqvist et al. 2016 [27]	1	1	0	1	1	1	1	1	1	8
Manikkam and Burns 2012 [28]	1	1	0	1	1	1	1	1	1	8
Manongi et al. 2017 [29]	1	1	0	1	1	0	1	1	0	6
Mokhele et al. 2019 [41]	1	1	0	1	1	1	1	1	1	8
Natamba et al. 2014 [30]	1	0	0	1	1	1	1	1	1	7
Nydoo et al. 2017 [20]	1	0	0	1	1	0	1	1	1	6
Okronipa et al. 2012 [42]	1	1	0	1	1	1	1	1	1	8
Osok et al. 2018 [21]	1	1	0	0	1	0	1	1	1	6
Rochat et al. 2011 [31]	1	0	0	1	1	1	1	1	1	7
Rubin et al. 2011 [32]	1	0	0	1	1	0	1	1	1	6
Stewart et al. 2008 [43]	1	1	0	1	1	1	1	1	0	7
Stranix-Chibanda et al. 2005 [33]	1	0	0	1	1	1	1	1	0	6
Thomas et al. 2017 [34]	1	1	0	1	1	0	1	1	1	7
Tomlinson et al. 2018 [35]	1	1	1	1	1	1	1	1	1	9

### Meta-analysis of antenatal depression

**Fig. 2:**
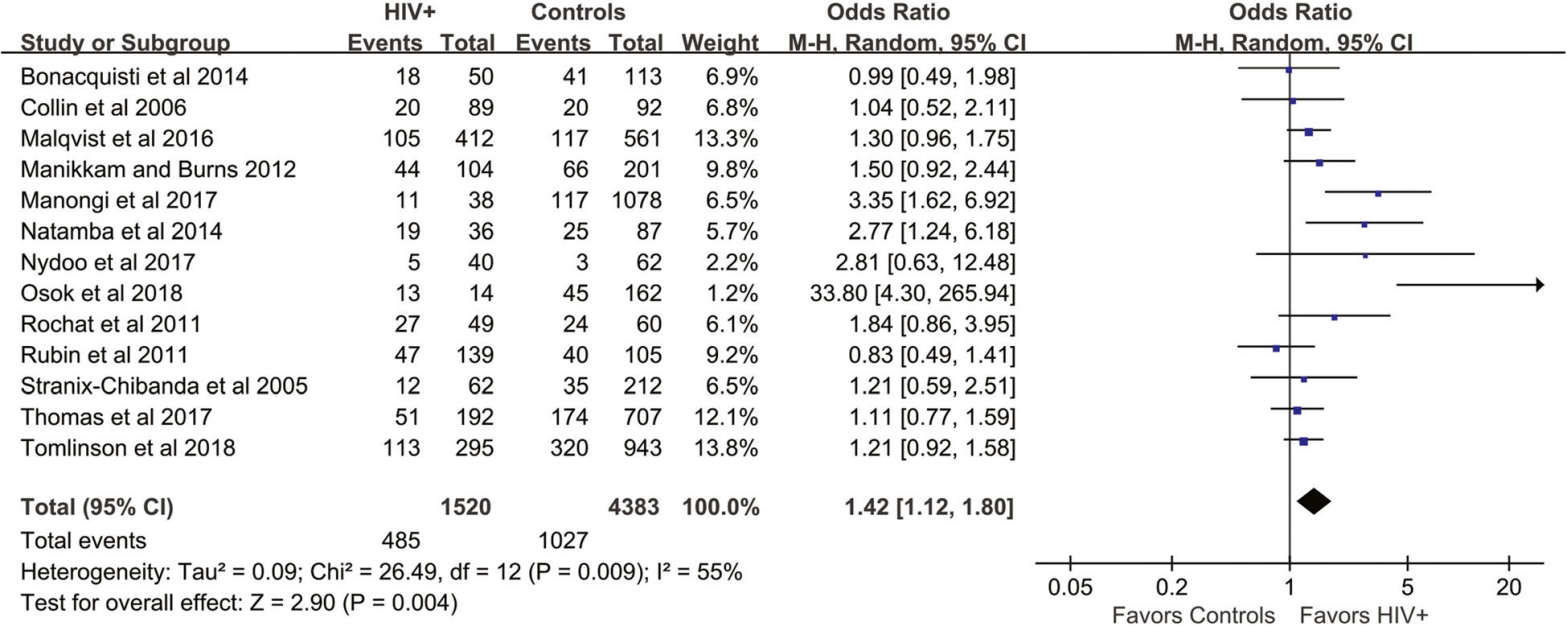
Forest plot of all included studies of antenatal depression in HIV-infected women compare with controls

The mean prevalence of antenatal depressive symptoms in thirteen included studies was 36% (95% CI: 27, 45%) in the HIV-positive group and 26% (95% CI: 20, 32%) in the control group. The meta-analysis showed a significantly increased odds ratio (OR) of antenatal depressive symptoms in the HIV infection group compared with controls (OR: 1.42; 95% CI: 1.12, 1.80), with moderate heterogeneity *I*^2^ = 55%, *P* = 0.004 (Fig. 2). The fixed-effects model showed a pooled OR of 1.32 (95% CI: 1.15, 1.52). When we excluded the study that used individuals with unknown HIV serostatus as controls, the result did not change significantly (OR: 1.44; 95% CI: 1.12, 1.86, random-effects model).

**Fig. 3:**
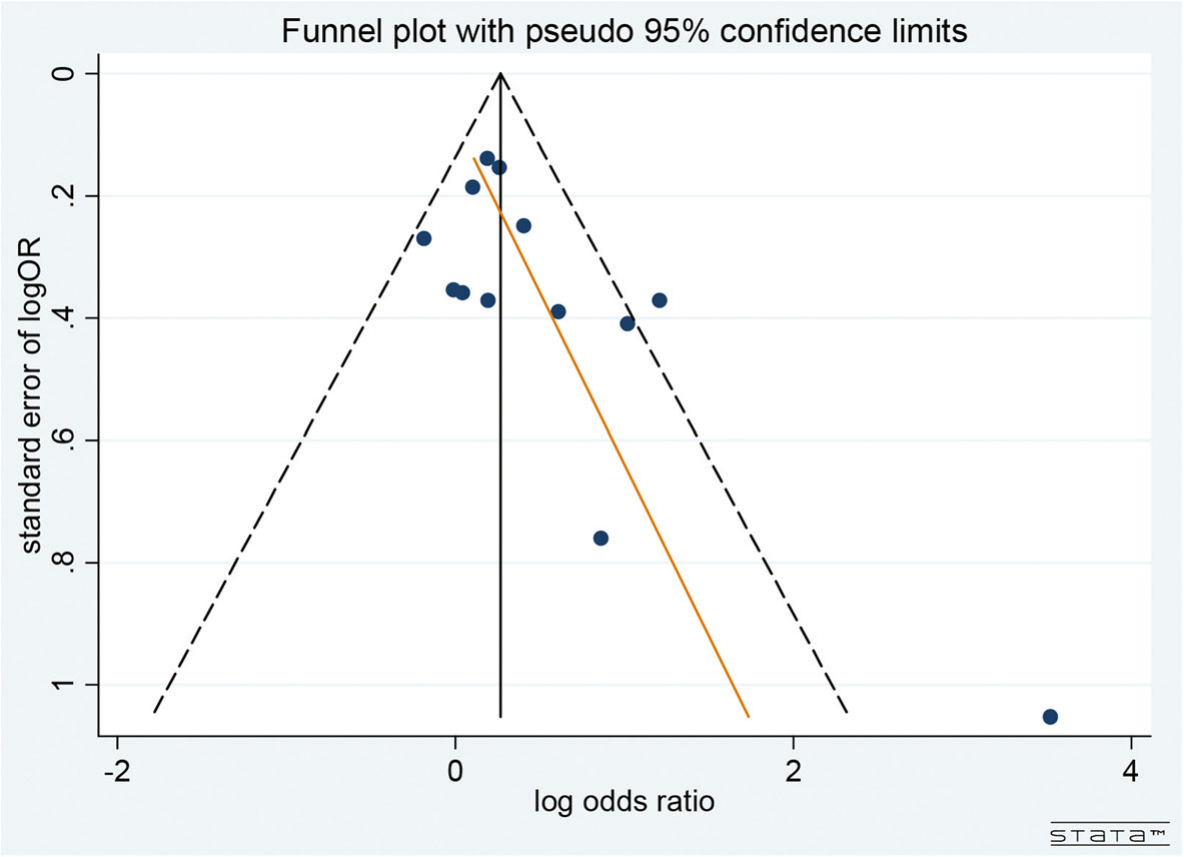
Funnel plot to assess for publication bias in antenatal studies

Our pre-specified sensitivity analyses are reported in Table 3. There were no differences among the subgroups when stratified by any methodological characteristics except the geographical location where the study was conducted; the studies that were conducted in Africa showed a higher OR than studies conducted in North America (*P* = 0.03). A funnel plot indicated the existence of publication bias, as a few included studies had a small sample size and reported negative findings (Fig. 3).

**Table 3:** Sensitivity analyses of methodological characteristics

Antenatal depression						Postnatal depression				
	Number of studies	Total number of subjects (HIV+/controls)	OR (95% CI)	*I*^2^ (%)	*P*	Number of studies	Total number of subjects (HIV+/controls)	OR (95% CI)	*I*^2^ (%)	*P*
Meta-analysis										
Random-effects model	13	1520/4383	1.42 (1.12, 1.80)	55		10	1645/2135	1.58 (1.08, 2.32)	65	
Fixed-effects model	13	1520/4383	1.32 (1.15, 1.52)	55		10	1645/2135	1.39 (1.13, 1.71)	65	
Sub group analysis										
Methodological quality					0.31					0.04
Low	0					0				
Medium	6	343/1732	1.88 (0.93, 3.79)	76		2	477/259	0.97 (0.64, 1.46)	0	
High	7	1177/2651	1.29 (1.11, 1.51)	0		8	1168/1876	1.86 (1.16, 2.98)	67	
Study design					0.11					0.13
Cross-sectional	8	808/2428	1.74 (1.19, 2.54)	58		7	1119/1763	1.92 (1.11, 3.34)	72	
Case-control	2	86/200	1.62 (0.59, 4.45)	73		1	49/113	1.55 (0.73, 3.30)		
Other	3	626/1755	1.11 (0.91, 1.36)	0		2	477/259	0.97 (0.64, 1.46)	0	
Depression screening tool					0.71					0.04
EPDS	7	1095/3714	1.58 (1.13, 2.21)	67		4	567/661	2.31 (1.17, 4.56)	69	
CES-D	3	225/305	1.25 (0.63, 2.47)	68		3	878/679	0.97 (0.72, 1.29)	0	
Other	3	200/364	1.30 (0.86, 1.99)	0		3	200/795	1.73 (0.82, 3.65)	32	
Geographical locations					0.03					0.19
Africa	11	1331/4165	1.55 (1.20, 2.01)	56		8	1457/1917	1.78 (1.12, 2.84)	68	
North America	2	189/218	0.88 (0.58, 1.35)	0		2	188/218	1.07 (0.58, 1.97)		

*Abbreviations*: *CES-D* Centre for Epidemiologic Surveys for Depression, *EPDS* Edinburgh Postnatal Depression Scale

### Meta-analysis of postnatal depression

**Fig. 4:**
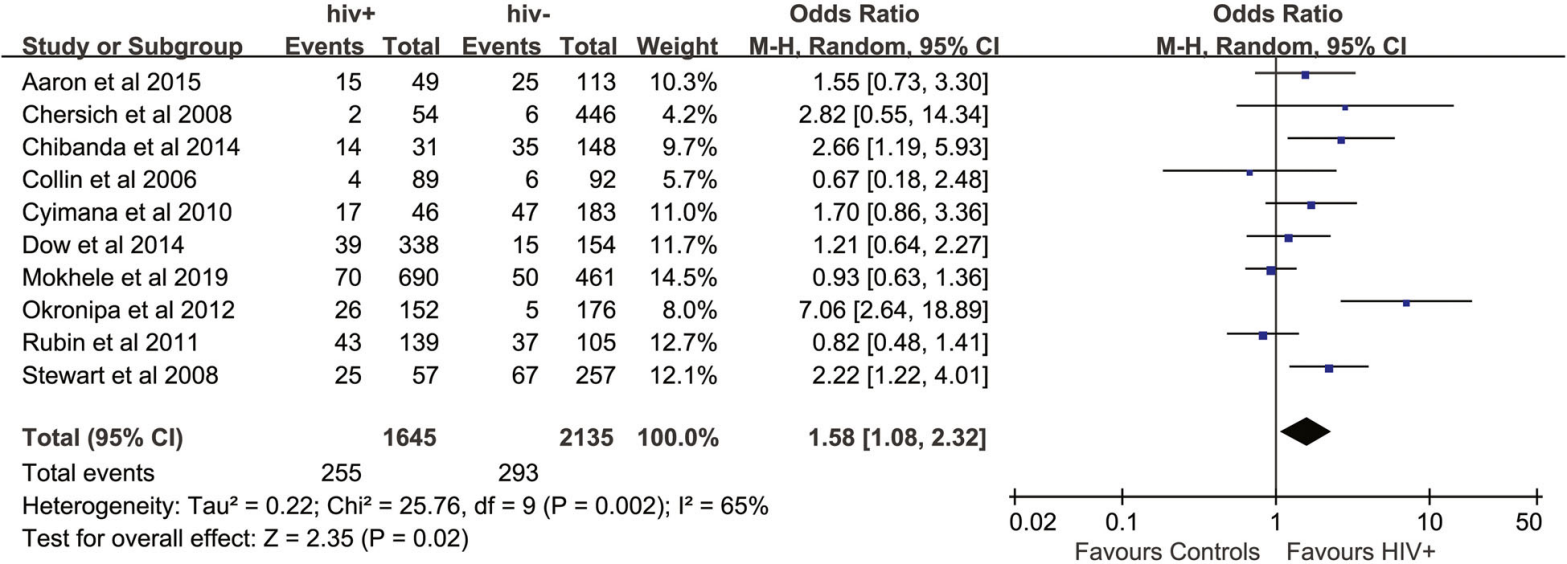
Forest plot of all included studies of postnatal depression in HIV-infected women compare with controls

The mean prevalence of postnatal depressive symptoms in ten included studies was 21% (95% CI: 14, 27%) in the HIV-positive group and 16% (95% CI: 10, 22%) in the control group. The meta-analysis showed a significantly increased odds ratio of postnatal depressive symptoms in the HIV-infected group compared with the controls (OR: 1.58; 95% CI: 1.08, 2.32), with moderate heterogeneity *I*^2^ = 65%, *P* = 0.002 (Fig. 4). The fixed-effects model showed a pooled OR of 1.39 (95% CI: 1.13, 1.71). However, when we excluded studies that used the EPDS with a cut-off value below the recommended 13, the odds ratio changed to 1.56 (95% CI: 0.98, 2.47).

**Fig. 5:**
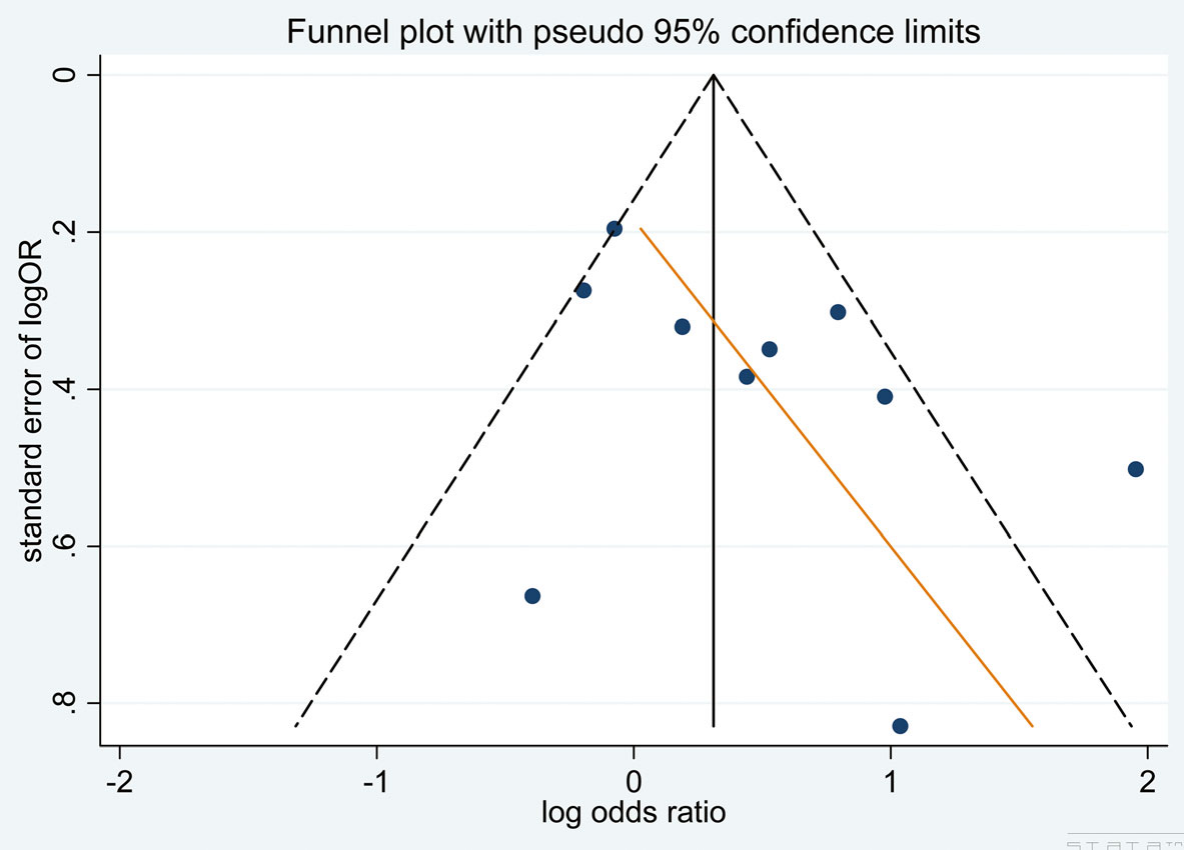
Funnel plot to assess for publication bias in postnatal studies

The pre-specified sensitivity analyses are reported in Table 3 and Additional file 2: Figures S5-S8. Differences were found among subgroups based on methodological quality, depression screening tool, and geographical locations. The high-quality studies showed a higher OR than the medium-quality studies (*P* < 0.01), and statistically significant differences were found among the studies with different study designs, including cross-sectional, case-control and longitudinal study (*P* < 0.01). The studies that were conducted in Africa showed a higher OR than those conducted in North America (*P* = 0.01). A funnel plot showed the existence of publication bias as a few included studies had a small sample size and reported negative findings (Fig. 5).

## Discussion

While several reviews have discussed the relationship between HIV infection and depression [44–46] and two reviews focused on the perinatal mental health of women with HIV infection [8, 47], more specific aspects made the present systematic review more unique. The disease burden of antenatal and postnatal depression among HIV-infected women could provide reference to develop appropriate screening programs and targeted interventions to reduce the negative health outcomes for the mothers and their babies. To our knowledge, this is the first meta-analysis to compare the prevalence of antenatal and postnatal depressive symptoms among HIV-infected women and controls. Our meta-analysis, which consisted of 23 studies, suggested that women living with HIV had a higher odds ratio of antenatal and postnatal depression compared with controls. This result is in accordance with several previous studies that suggested a high prevalence of antenatal and postnatal depression among HIV-infected women [48, 49].

For perinatal depression, depressive symptoms were measured with different screening tools. The most commonly used measurement tool was the EPDS; eleven of the 23 included studies used it in our meta-analysis. The present meta-analysis included studies with different measurement tools for perinatal depressive symptoms, even the same measurement tool with different cut-off values. As we shown in our results, when we excluded the studies of which screening tool EPDS cut-off values were below 13, the odds ratio varied from 1.58 (95% CI: 1.08, 2.32) to 1.56 (95% CI: 0.98, 2.47). Thus, it was indicated that the adoption of screening tools for depressive symptoms and the choice of cut-off value should be considered in the meta-analysis that including studies with different screening tools.

While the prevalence of antenatal and postnatal depression in HIV-infected women was modestly higher than that in controls, significant clinical effects may be associated with this difference. Several studies have focused on the complexity of the causal pathways between HIV infection and depression, and extensive evidence has been found that the persistent existence of the virus in the central nervous system could result in neurobiological changes that might cause depression in HIV-infected individuals [50–55]. Del Guerra et al. conducted a review to examine the key role of neuroendocrine, immunoinflammatory, and monoaminergic mechanisms in the development of depression among HIV-infected patients [50]. Few studies have focused on the mechanisms of depression in pregnant women with HIV, and it is of great significance to study the effects of HIV infection on pregnant women and their offspring. The mean prevalence of antenatal depression in HIV-positive and controls was 36 and 26%, respectively, which was relatively high. This could be partly explained by the fact that the depressive symptoms were measured based on various screening tools, and this also drew the attention to the depression during antenatal care. A systematic review by Sowa et al. has reported a prevalence of 43.5% of susceptible antenatal depression among HIV-positive women, which is similar to ours [8].

Furthermore, antenatal depression might be a risk factor for postnatal depression [56]. Verreault et al. found that among women with postnatal depression, most have experienced depression during pregnancy, and only 6.6% were new cases [57]. While adverse foetal and maternal outcomes and non-adherence to ART and prenatal care are associated with untreated perinatal depression [5, 12–19], some studies proved that both maternal and foetal physical health and psychosocial outcomes can be improved with proper intervention [58]. Therefore, it is imperative to improve prenatal care and ART adherence among HIV-infected women. A meta-analysis revealed that ART adherence was improved with the application of treatments for depression and psychological distress [59]. According to our results that in the HIV-positive group the prevalence of antenatal and postnatal depressive symptoms was 36 and 21%, respectively, which was relatively high, thus it is imperative to take action in this particular population.

Gestation is associated with diverse physiological changes and can be regarded as the most effective period for depression screening and treatment. Given the large number of HIV-infected women, it is a major challenge for local medical systems to meet their prenatal health needs by offering both medical and psychological interventions. Regular prenatal care not only protects women’s physical and psychological health in the postpartum but also decreases the mothers’ risk to have low birth weight, preterm, and small for gestational age babies [60]. The depression rate is also significantly decreased among HIV-infected individuals who receive social support [61]. Some studies have confirmed that adherence to ART decreased during the postpartum period when antenatal depression persisted after delivery [62–64]. In addition, improved prenatal psychological health may contribute to postpartum emotional adjustment, thus helping to establish a good maternal–child bond, enhancing the mother’s social skills and expanding her social support network in a virtuous circle. For perinatal depression, available interventions found to be efficacious generally consist of cognitive-behavioural and interpersonal therapy approaches [65, 66]; and among them, for example, the Mothers and Babies course is a cognitive-behavioural intervention that has shown promise as an intervention for low-income women at risk for postnatal mood issues [67]. The intervention during the antenatal and postnatal period need to be further explored and assessed, the optimal management of comorbid HIV and depression should also be further investigated.

There are some strengths in this study. First, it is the first meta-analysis to compare the prevalence of perinatal depression symptoms between HIV-infected women and controls, which could add to guidelines for the treatment of this population. Second, the synthesis and analysis of a large number of studies from countries in Africa with a high HIV prevalence suggests a higher risk of both antenatal and postnatal depression among women living with HIV infection, adding evidence to previous articles that focused on the high prevalence of depression among HIV-positive women [8, 46]. Because the prevalence of antenatal and postnatal depression rather than mean scores could be obtained from the included studies, mean prevalence of antenatal and postnatal depressive symptoms could be calculated, and thus we found that prevalence of antenatal depressive symptoms was much higher than that of postnatal depressive symptoms. This might be partly explained that the characteristics of the populations in the two different sets of publications were different. And this also implies that there may be a biological relationship between HIV and depression that is seen in the biological environment of pregnancy but not postpartum, which merits further attention and investigation.

Nevertheless, several limitations exist in this study. First, screening tools were used to measure depressive symptoms rather than depression, and the same cut-off values of each screening tool were not used in all studies, so we should cautiously apply the results to the population. Second, because the baseline of each study was not exactly the same and individual information was not available, and the heterogeneity cannot be fully explained by subgroup analysis. Third, six types of screening tools were used to detect perinatal depression. While the EPDS was designed specifically for pregnant women, the application of other tools might not be suitable and might have contributed to the heterogeneity. Fourth, the studies that we included had a variety of study designs, which might be another contributor to the heterogeneity.

## Conclusions

Our meta-analysis demonstrates a significantly increased risk of antenatal and postnatal depressive symptoms in women with HIV infection, and the findings emphasize the need for the optimal management of comorbid HIV and depression. Future studies should focus on the longitudinal follow-up of women with HIV infection, the causal pathways between HIV and perinatal depression and the long-term impact of HIV-related treatments on antenatal and postnatal depression in this high-risk population.

## Supplementary information

**Additional file 1.** PRISMA checklist

**Additional file 2.** Supplemental Figures for subgroup analyses

## Data Availability

All data generated or analyzed during this study are included in this published article.

## Abbreviations

ART: Antiretroviral therapy
CES-D: Centre for Epidemiologic Surveys for Depression
CI: Confidence interval
DSM-5: The Diagnostic and Statistical Manual of Mental Disorders Fifth Edition
EPDS: Edinburgh Postnatal Depression Scale
HIV: Human immunodeficiency virus
ICD-10: International Classification of Diseases 10th Revision
MDE: Major depressive episode
OR: Odds ratio
RevMan: Review Manager
SCID: Diagnostic Structured Clinical Interview for DSM-IV Axis I Disorders
SRQ: Self-Reporting Questionnaire
