# Supplementary material for: Prevalence of perinatal depression among HIV-positive women: a systematic review and meta-analysis
Source: BMC Psychiatry. 2019 Oct 30;19:330. doi: 10.1186/s12888-019-2321-2 (PMC6822469; doi:10.1186/s12888-019-2321-2)
Supplement: Supplementary file 2 — Additional file 2. Supplemental Figures for subgroup analyses [file 12888_2019_2321_MOESM2_ESM.docx]

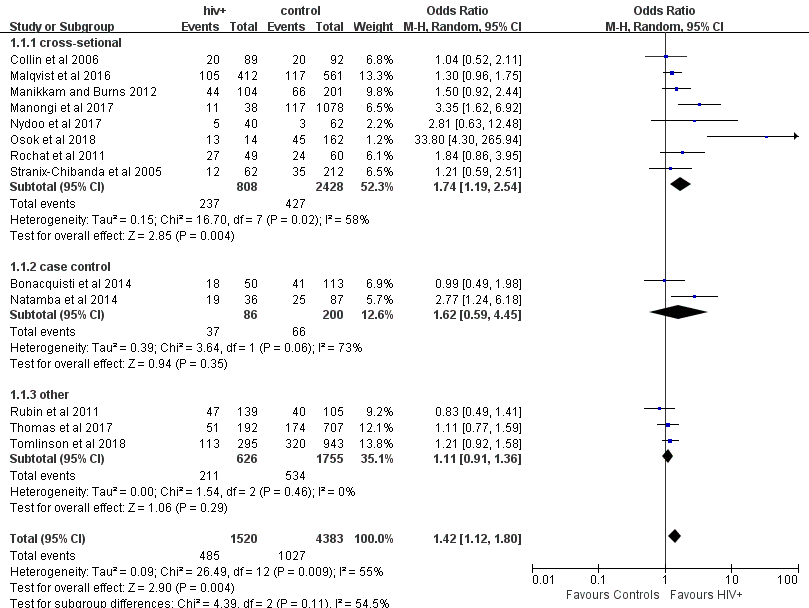


**Fig. S1.** Sub-group analysis of different study designs of antenatal depression in HIV-infected women compare with controls


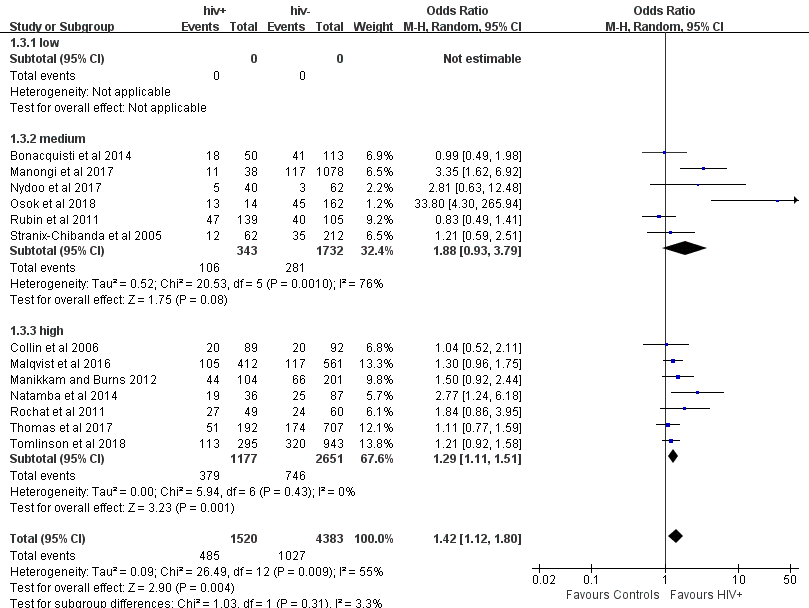


**Fig. S2.** Sub-group analysis of different study quality of antenatal depression in HIV-infected women compare with controls


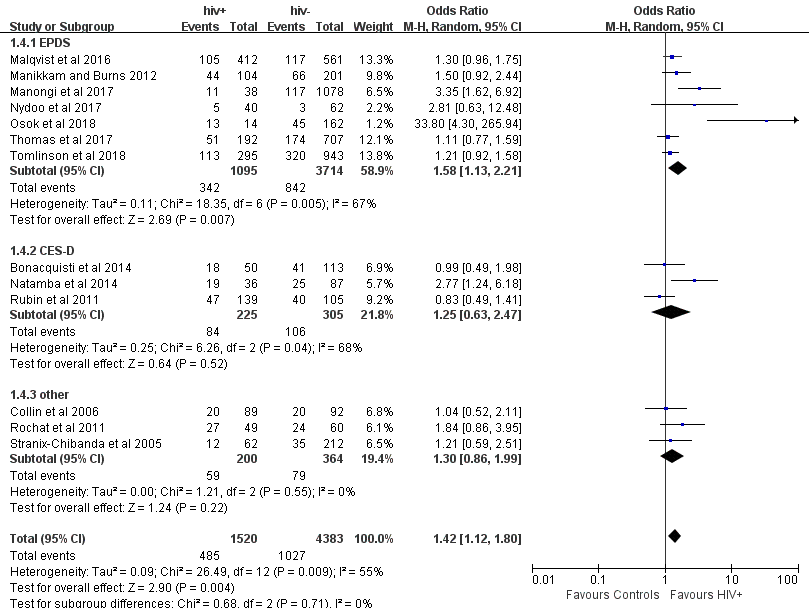


**Fig. S3.** Sub-group analysis of different screening tools of antenatal depression in HIV-infected women compare with controls


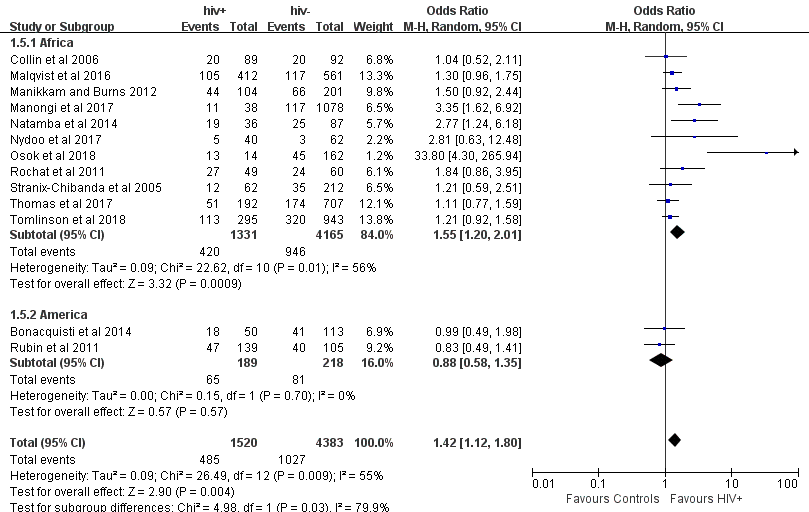


**Fig. S4.** Sub-group analysis of different geographical locations of antenatal depression in HIV-infected women compare with controls


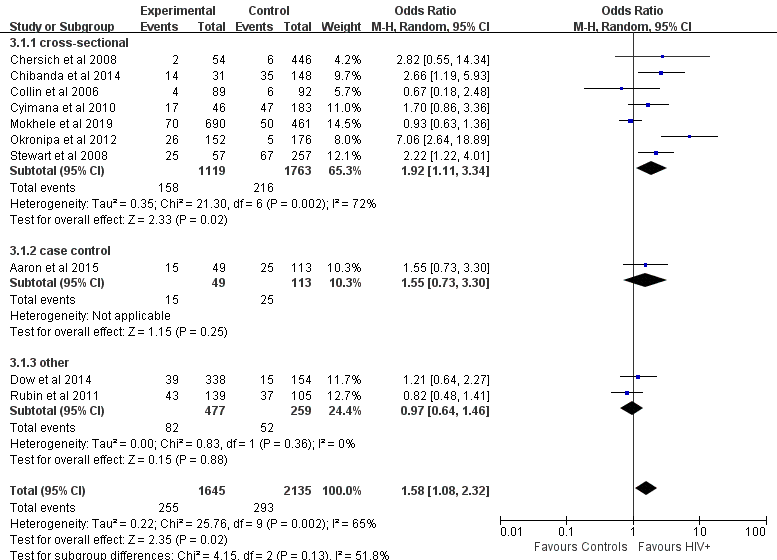


**Fig. S5.** Sub-group analysis of different study designs of postnatal depression in HIV-infected women compare with controls


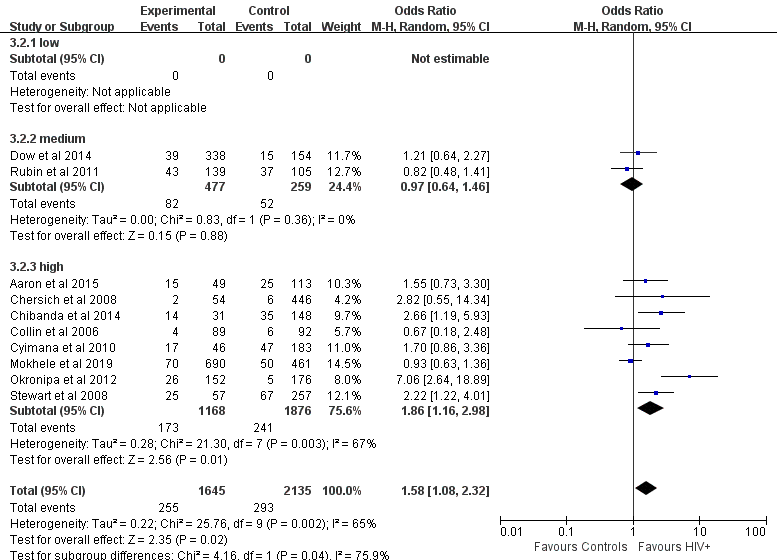


**Fig. S6**. Sub-group analysis of different study quality of postnatal depression in HIV-infected women compare with controls


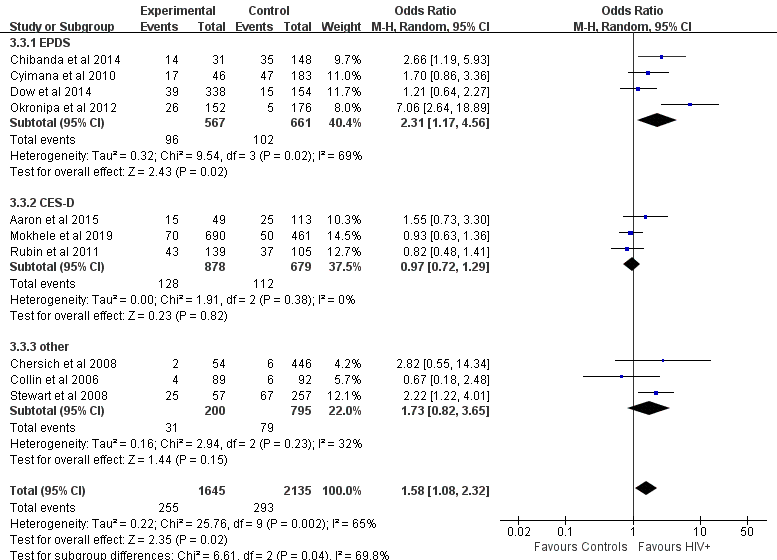


**Fig. S7.** Sub-group analysis of different screening tools of postnatal depression in HIV-infected women compare with controls


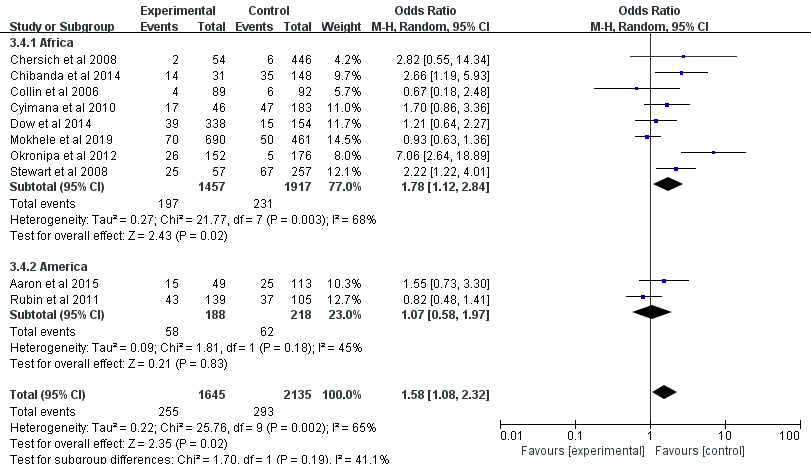


**Fig. S8.** Sub-group analysis of different geographical locations of postnatal depression in HIV-infected women compare with controls
